# Antimicrobial Susceptibility of Enterotoxigenic *Escherichia coli* from Diarrhoeic Neonatal Calves in Spain

**DOI:** 10.3390/ani12030264

**Published:** 2022-01-21

**Authors:** Alberto Prieto, Cynthia López-Novo, Pablo Díaz, José Manuel Díaz-Cao, Gonzalo López-Lorenzo, Claudia Antón, Susana Remesar, David García-Dios, Ceferino López, Rosario Panadero, Pablo Díez-Baños, Patrocinio Morrondo, Gonzalo Fernández

**Affiliations:** INVESAGA Group, Departamento de Patoloxía Animal, Universidade de Santiago de Compostela, 27002 Lugo, Spain; alberto.prieto@usc.es (A.P.); pablo.diaz@usc.es (P.D.); josemanueldiaz.cao@usc.es (J.M.D.-C.); gonzalo.lopez.lorenzo@gmail.com (G.L.-L.); claudia.anton.fdez@gmail.com (C.A.); susana.remesar@usc.es (S.R.); david.garcia.dios@rai.usc.es (D.G.-D.); c.lopez@usc.es (C.L.); rosario.panadero@usc.es (R.P.); pablo.diez@usc.es (P.D.-B.); patrocinio.morrondo@usc.es (P.M.); gonzalo.fernandez@usc.es (G.F.)

**Keywords:** neonatal calf diarrhoea, *Escherichia coli*, ETEC, antimicrobial susceptibility

## Abstract

**Simple Summary:**

Neonatal calf diarrhoea, a worldwide concern for cattle production, can be caused by viral, bacterial and protozoan enteropathogens: the enterotoxigenic *Escherichia coli* (ETEC) is one of the most important. The use of antimicrobials for treating neonatal calf diarrhoea cases is still a common practice among veterinary surgeons, although its use is only justified in bacterial infections evolving towards a systemic disease. Since the indiscriminate use of antimicrobials for treating diarrhoeic calves increases the risk for the appearance of antimicrobial resistances, restrictions on the use of antimicrobials in veterinary practice were implemented. The aim of this study was to characterize the antimicrobial susceptibility of ETEC strains obtained from diarrhoeic calves. Our results are alarming since all ETEC strains were resistant to three or more families of antimicrobials; in addition, a high number of strains were resistant to most first-line antimicrobials used in veterinary practice. Only ceftiofur, cefoperazone, cefquinome and gentamicin presented efficacy against most ETEC strains. Thus, empirical treatment of calf scours caused by ETEC is usually ineffective. Our results reveal that performing antimicrobial susceptibility tests in each NCD outbreak is needed for establishing an effective treatment and avoiding the emergence of new resistance mechanisms.

**Abstract:**

Enterotoxigenic *Escherichia coli* (ETEC) is one of the major pathogens involved in neonatal calf diarrhoea (NCD) causing high economic losses in dairy farms. Antibiotic treatment is common in cases of systemic illness caused by NCD, but antimicrobial susceptibility tests (AST) are usually not performed. Thus, the aim of this study was to characterize the antimicrobial susceptibility of ETEC strains obtained from calves with diarrhoea between 2018–2020. Faecal samples (*n* = 420) were analyzed to detect the typical ETEC virulence factors F5 and STa. Positive samples were cultured to identify and isolate ETEC strains (*n* = 41) and ASTs were performed. Our results are alarming since ETEC strains resistant to three or more families of antimicrobials were detected in all isolates. Only four antibiotics (ceftiofur, cefoperazone, cefquinome and gentamicin) presented efficacy against more than 90% of the ETEC strains, while the other ten antibiotics were effective against less than 40% of the strains. In addition, a high number of strains were resistant to most first-line antimicrobials used in veterinary practice. For this reason, when ETEC infection is suspected, an AST must always be performed to select the most appropriate antimicrobial in each case and to avoid the emergence of new resistance mechanisms.

## 1. Introduction

Neonatal calf diarrhoea (NCD) is probably the major cause of mortality in calves younger than one month, becoming a worldwide concern for dairy and beef cattle production [1,2]. A wide variety of viral, bacterial and protozoan enteropathogens can be involved in the onset of this process, and coinfections are usually observed; nevertheless, some of them are considered as primary agents that can produce NCD in the absence of other enteropathogens [3,4,5]. Among these primary pathogens, one of the most important is enterotoxigenic *Escherichia coli* (ETEC) [3,6,7]. This *E. coli* pathotype is characterized by presenting different specific adhesins and enterotoxins, being the combination of F5 fimbriae and STa heat-stable enterotoxin the most frequent in cattle strains [7]; these virulence factors are responsible for the colonization of the intestinal mucosa and the osmotic disequilibrium, which finally leads to the development of diarrhoea. ETEC usually affects calves in their first few days of life, inducing a mild to per-acute watery diarrhoea that can also be accompanied by systemic illness [8].

The application of antibiotic therapy for treating ETEC infections remains a controversial topic. In this respect, most authors only justify its use in cases which are evolving towards a systemic disease, for both preventing bacteriemia and reducing the number of ETEC in the gut [9,10,11]. However, the use of antimicrobials for treating NCD is still a common practice among veterinary surgeons, even though ETEC involvement has not been demonstrated. In fact, a recent survey performed in four European countries revealed that 52.5% of farmers and veterinarians used antimicrobials in diarrhoeic neonatal calves, and at least 27% of these respondents used them for treating this syndrome in all cases [12]. In the present framework of increasing emergence of multi-resistant bacteria, the rational use of antibiotics has arisen as a global concern for the last decade; for this reason, the World Health Organization fosters the Global Antimicrobial Resistance and Use Surveillance System (GLASS) for developing strategies to contain antimicrobial resistance [13]. In Europe, some of these approaches have resulted in restrictions on the use of antimicrobials in veterinary practice, banning some antibiotic families for animal productions and limiting others based on the results of antimicrobial susceptibility testing (AST) [14]. As a result of these policies, veterinary practitioners have fewer therapeutical options for the empirical treatment of bacterial infections, including ETEC diarrhoea. In this context, the knowledge of how antibiotic resistances are evolving in veterinary medicine is essential for optimizing the selection of antibiotics and improving their use in animal production.

For the aforementioned reasons, the aim of this work was to characterize the phenotypical antimicrobial susceptibility of ETEC strains isolated from NCD cases in Spain against the main antimicrobial families used for treating this pathogen in veterinary medicine. The results will allow determining the presence and frequency of the different antimicrobial resistances, and thus providing updated and useful information for the treatment of this syndrome.

## 2. Materials and Methods

### 2.1. Sample Collection and Primary Isolation of E. coli

From 2018 to 2020, 420 diarrhoeic calves under one month of age were sampled by veterinary practitioners in 222 cattle farms from NW Spain, a region that gathers a quarter of Spanish cattle. Faecal samples were collected directly from the rectum using a swab with Amies transport medium (Deltalab, Barcelona, Spain). All swabs were kept refrigerated and were submitted to the laboratory in the 24 h post-collection. Swabs were then plated onto MacConkey agar plates (VWR Chemicals, Leuven, Belgium) and incubated at 37 °C overnight. Three lactose-positive compatible colonies from each agar plate were isolated and identified biochemically with an API system (API 20E, bioMérieux, Marcy l’Etoile, France) to confirm the isolation of *E. coli* strains.

### 2.2. Identification of ETEC Strains

Those isolates, confirmed biochemically as *E. coli*, were screened by qPCR for detecting DNA coding the production of STa enterotoxin and F5 fimbriae, the typical virulence factors of bovine ETEC strains. Firstly, each *E. coli* isolate was plated in sheep-blood agar (VWR Chemicals, Leuven, Belgium) and incubated at 37 °C overnight. Total DNA was extracted taking a loopful of bacterial growth from each isolate, which was suspended in a 1.5 mL microtube containing 200 μL of Tris-EDTA buffer (Sigma-Aldrich, St. Louis, MO, USA). The tubes were incubated at 100 °C for 10 min and subsequently placed on ice for another 10 min. After centrifugation of lysates at 12,000× *g* for 5 min, 100 μL of supernatant was transferred to a clean microtube and kept at −80 °C until qPCR analysis.

DNA samples were analyzed using two commercial qPCR kits, one targeting the *f5* gene and another targeting the *sta*, *stb* and *lt* genes (EXOone *E. coli* virulence factor F5 (K99) and EXOone *E. coli* Sta—STb—LT, Exopol SL, Zaragoza, Spain) following the manufacturer’s instructions. Specific primers and probe for the *gadAB* gene of *E. coli* were also used as an internal positive control, allowing the detection of possible qPCR inhibition. In each run, synthetic positive controls supplied by the manufacturer and molecular grade water were employed as positive and negative controls, respectively. Reactions were run on an Applied Biosystems ABI Prism 7500 thermocycler (Thermo Fisher Scientific, Waltham, MA, USA). All positive samples for both the *f5* and *sta* genes were considered as ETEC. The corresponding isolates were stored in glycerol stock solution at −80 °C.

### 2.3. Antimicrobial Susceptibility Test

Confirmed ETEC isolates (a single ETEC strain randomly selected among all compatible strains from each faecal sample) were cultured in sheep-blood agar, and two or three colonies were suspended in 2 mL of sterile saline solution to achieve a McFarland turbidity ranging from 0.55 to 0.62. The ASTs were subsequently performed with the automated VITEK-2 system using specific veterinary AST-GN96 cards for Gram-negative bacteria (bioMérieux, Marcy l’Etoile, France) and following the manufacturer’s instructions. This card analyzes fourteen antimicrobials belonging to five different antimicrobial families (beta-lactams, aminoglycosides, quinolones, tetracyclines and trimethoprim/sulphonamides), and it also includes a test for identifying extended spectrum β-lactamase (ESBL)-producing strains. The results of minimum inhibitory concentration (MIC) for each antibiotic were used for classifying ETEC isolates as Susceptible (S), Intermediate (I) or Resistant (R) according to international standards. In addition, the MIC_90_ (MIC required to inhibit the growth of 90% of isolates) were also calculated. The antimicrobials tested as well as MIC breakpoints and their standards are summarized in Table 1.

## 3. Results

**Figure 1:**
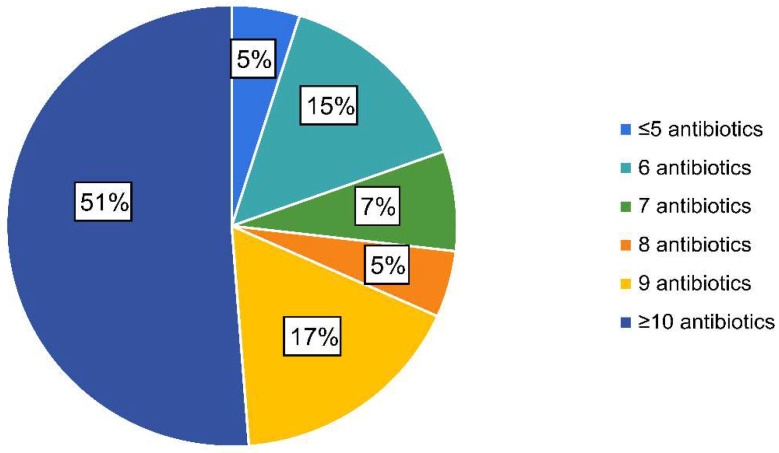
Percentage of enterotoxigenic *Escherichia coli* isolates considering the number of antimicrobial resistances per isolate.

Analysis of qPCR results allowed the identification of ETEC isolates in 41 (9.8%) out of the 420 diarrhoeic faecal samples; a complete AST was obtained for all of them. All the strains were resistant to more than four antimicrobials and 51% (*n* = 21) to ten or more out of the fourteen tested (Figure 1). The most common resistance combination (44%) (*n* = 18) included ampicillin, amoxicillin/clavulanic acid, cefalexin, cephalothin, neomycin, flumequine, enrofloxacin, marbofloxacin, tetracycline and trimethoprim/sulfamethoxazole. In addition, it must be noticed that ≈5% (*n* = 2) of the strains were identified as ESBL-producing strains.

**Figure 2:**
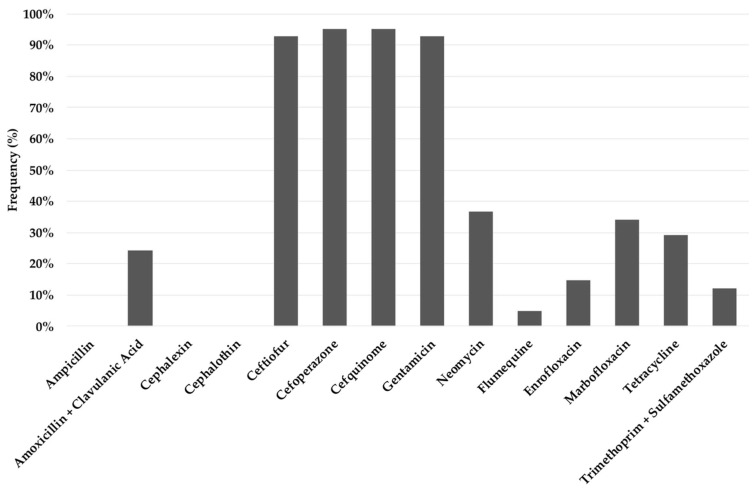
Percentage of enterotoxigenic *Escherichia coli* isolates susceptible to each antimicrobial tested.

Noticeable differences in the number of ETEC isolates resistant to each antibiotic were found (Figure 2). Thus, resistance to ampicillin, cephalothin and cephalexin was detected in all strains; in contrast, the most effective antibiotics were cefoperazone/cefquinome and ceftiofur/gentamicin since 95% and the 93% of the isolates, respectively, were susceptible. A low percentage (4–37%) of susceptible strains against the remaining seven antibiotics was also found. When considering the five antimicrobial families, all the isolates resulted resistant to at least one antibiotic from three families, coinciding with the definition of multidrug-resistant (MDR) strains [15].

**Figure 3:**
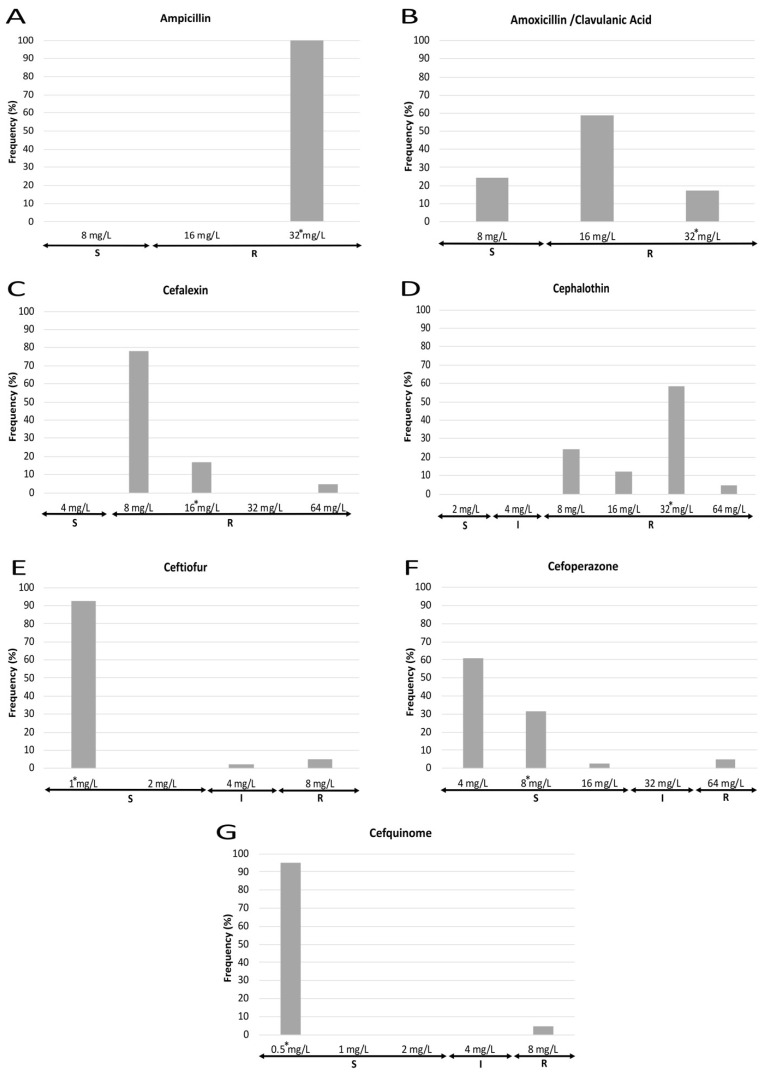
MIC frequencies for the different beta-lactams tested: ampicillin (**A**), amoxicillin/clavulanic acid (**B**), cefalexin (**C**), cephalothin (**D**), ceftiofur (**E**), cefoperazone (**F**) and cefquinome (**G**). The asterisk (*) indicates the MIC_90_. S: susceptible (standard dose); I: intermediate (susceptible at increased exposure); R: resistant.

**Figure 4:**
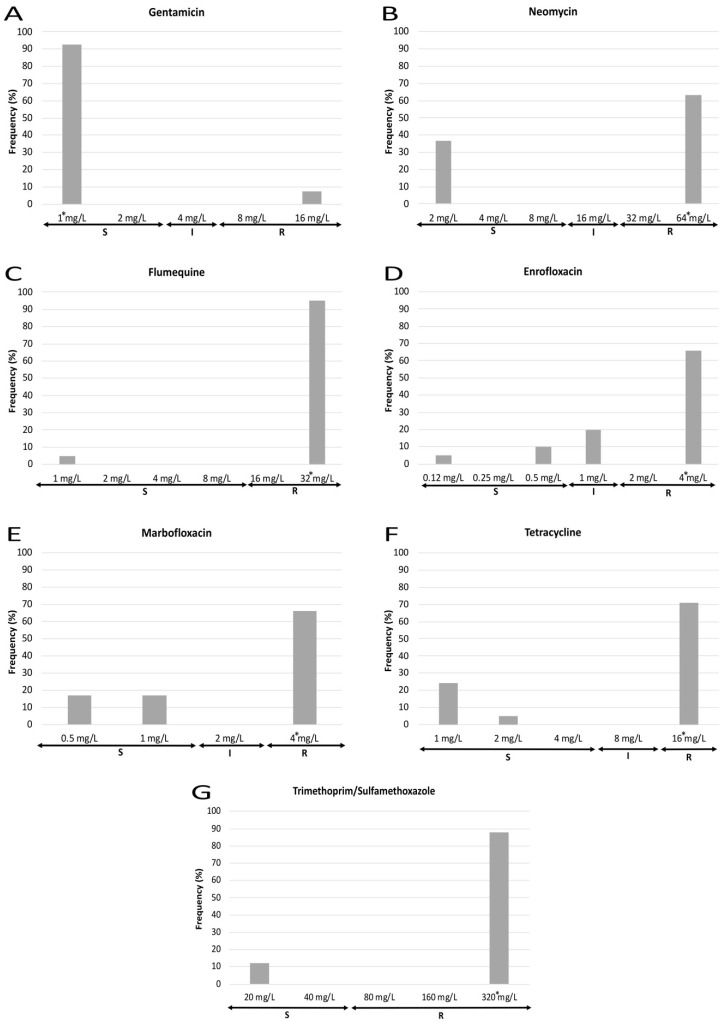
MIC frequencies for gentamicin (**A**), neomycin (**B**), flumequine (**C**), enrofloxacin (**D**), marbofloxacin (**E**), tetracycline (**F**) and trimethoprim/sulfamethoxazole (**G**). The asterisk (*) indicates the MIC_90_. S: susceptible (standard dose); I: intermediate (susceptible at increased exposure); R: resistant.

MIC results also show marked differences in the susceptibility to each antibiotic tested (Figure 3 and Figure 4). Regarding those antibiotics to which all the strains were resistant, 100% of strains presented a MIC value for ampicillin equal to or higher than the maximum concentration of the antimicrobial employed, whereas cephalexin and cephalothin showed different MIC values dispersed along the resistant category. With respect to those antimicrobials to which most of the strains were resistant, three different patterns were observed: first, for amoxicillin/clavulanic acid, most isolates showed a MIC value near the susceptibility breakpoint; second, for fluoroquinolones (enrofloxacin, marbofloxacin), those strains showing a MIC value equal to the highest concentration tested were the most frequent (>60%), while a lower percentage of isolates were classified as susceptible and intermediate; and third, a polarized pattern was observed for flumequine, neomycin, tetracycline and trimethoprim/sulfamethoxazole, with few isolates which were susceptible to the lowest concentration of the antimicrobial opposed to the rest which were resistant to the highest concentration. Finally, for ceftiofur, cefoperazone, cefquinome and gentamicin, most of the isolates were sensitive to the lowest concentration that was tested. Regarding the MIC_90_, it must be noticed that it was equal to the highest concentration used for eight out of fourteen antimicrobials.

## 4. Discussion

Although there is extensive scientific literature regarding antimicrobial resistance (AMR) in *E. coli*, reports on specific pathotypes involved in NCD are scarce. Thus, the present study is focused only in ETEC strains isolated from NCD cases, revealing that AMR is extremely common in this pathotype in calves from Spain. These results agree with previous data reported for other pathotypes in Spain and other countries, being the consequence of the huge genomic plasticity of *E. coli* and the frequent phenomena of co-transfer of AMR and virulence genes among pathogenic strains [16,17,18,19,20,21,22]. In addition, our results must be considered alarming since 100% of ETEC isolates were identified as MDR strains, and 44% were simultaneously resistant to tetracyclines, trimethoprim/sulphonamides, fluoroquinolones, and a number of beta-lactam antibiotics. 

Beta-lactam antibiotics represent one of the most recurring therapeutic options in veterinary practice, particularly aminopenicillins applied alone or combined with beta-lactamase inhibitors [23]. Extensive administration of these antimicrobials may be the major reason for the high percentage of ETEC strains resistant to ampicillin (100%) or amoxicillin/clavulanate (76%) detected in this study. These results are not surprising since previous worldwide investigations also report high prevalence of *E. coli* resistant strains from NCD cases [24,25,26,27]. Similarly, all ETEC strains resulted resistant to first-generation cephalosporins such as cefalexin and cephalothin; these results were higher than those previously reported in diarrhoeic calves from Iran, where 65.9% of ETEC isolates were resistant to cephalotin [26]. In contrast, third- and fourth-generation cephalosporins were very effective against ETEC since the percentage of susceptible isolates ranged from 93% to 95%. These findings are surely related to the different resistance mechanisms acquired by most of these strains, suggesting that hyperproduction of beta-lactamases such as TEM-1, TEM-2 and SHV-1 or inhibitor-resistant beta-lactamases such as IRT and OXA-1 could be implicated [28,29]. In addition, only a low number of ESBL-positive isolates were identified (≈5%), being consistent with most reports on NCD describing a low proportion or even no ESBL-producing strains [24,27,30,31]; however, there is a need for further monitoring the emergence of this resistance mechanism since it is a topic of concern from the One Health point of view.

Considerable differences in the AMR of the two aminoglycoside antibiotics tested were found since a very low proportion of strains showed resistance to gentamicin (7%) and a quite high proportion to neomycin (63%). Although, in general, these results agree with previous investigations [17,32,33], it is worth noting that substantial differences in the prevalence of aminoglycoside-resistant *E. coli* have been worldwide described in NCD studies [21,26,34,35]. This fact is probably related to diverse antimicrobial prescribing policies among countries as well as to the antimicrobial availability and legislation for restricted use of some antibiotics in veterinary practice [36,37]. Resistance to this antimicrobial family can occur through different mechanisms, but the enzymatic inactivation is the most common [38]. There are three major classes of enzymes modifying aminoglycosides (acetyltransferases -AACs-, nucleotidyltranferases -ANTs-, and phosphotransferases -APHs-) and their presence can be phenotypically inferred using different aminoglycosides [28,38,39]. Although only two aminoglycoside-antibiotics were tested in this study, our AMR results suggest that the most prevalent mechanisms of resistance were the production of APH(3′) and/or AAC(6′) since most ETEC isolates (61%) were gentamicin-sensitive and neomycin-resistant [28,40].

Our data also reveal that fluoroquinolones were one of the antimicrobial families showing the highest AMR rates since 95% and 66–85% of ETEC isolates were resistant to first- and third-generation fluoroquinolones, respectively. These are concerning results since the different international health organizations consider third- and fourth-generation fluoroquinolones as antibiotics of critical importance for human medicine [14,41]; nevertheless, these findings could be explained by the fact that fluoroquinolones are among the most used antimicrobials for treating NCD [12]. Resistance to quinolones relies mainly on accumulative mutations of their molecular targets (“quinolone resistant determining regions” -QRDR-) and/or acquisition of specific plasmids (“plasmid-mediated quinolone resistance” -PMQR-) [39,42]. Both mechanisms induce a progressive resistance to this antibiotic family; thus, those isolates resistant to first-generation analogues are more prone to develop reduced susceptibility or resistance to fluoroquinolones of subsequent generations [39,40,42,43]. This characteristic effect of fluoroquinolones, together with their abusive use, may be the cause of the high percentages of ETEC strains resistant to this antimicrobial family as well as the upward trend in MIC values for third-generation compounds.

Phenotypical susceptibility to tetracyclines and trimethoprim/sulphonamides is usually inferred from the result of a single class-representative antimicrobial within each family [40]; since the identification of particular mechanisms of resistance in both families is not possible using only the phenotypical inference, molecular characterization for establishing the specific genes involved is needed. Our data showed that both families presented high rates of resistant isolates (71% for tetracyclines and 88% for trimethoprim/sulphonamides) agreeing with previous investigations on *E. coli* strains from NCD, although most studies reported a higher percentage of isolates resistant to tetracyclines (75–100%) than to trimethoprim/sulphonamides (35–84%) [24,26,30,32,33,35]. In this study, the differences observed between both families may be related to a less use of tetracyclines compared to trimethoprim/sulphonamides for the treatment of NCD; in fact, recent data from European countries demonstrate that tetracyclines were used as first choice treatment in less than 15% of NCD cases whereas sulphonamides were chosen in more than 70% of cases [12].

NCD is, together with mastitis, one the main pathologies inducing AMR [44,45]. Although feeding calves with milk from dams receiving antibiotics have been considered as a risk for the appearance of AMR, the indiscriminate use of antimicrobials for NCD treatment, even if a bacterial aetiology has not been confirmed, has been suggested as the most probable reason [45,46,47]. Regarding the usual antimicrobial treatments proposed, reviews have focused on the treatment of NCD indicate aminopenicillins, third- and fourth-generation cephalosporins, trimethoprim/sulphonamides, tetracyclines and, in some cases, fluoroquinolones as the most recommended antimicrobials for ETEC infections; nevertheless, most of these authors suggest that antimicrobial therapy must only be used in those cases showing systemic involvement [10,48,49,50]. Regrettably, the latest studies show that antibiotics are still used indiscriminately in treating this condition regardless of the aetiology or presentation [12]. In addition, our results reveal that only gentamicin and third- and fourth-generation cephalosporins can be considered as an effective therapeutic option for ETEC infections causing NCD since most isolates were susceptible. However, these antimicrobials are listed as Category C (gentamicin) or even Category B (third- and fourth-generation cephalosporins) according to the recommendations of the Antimicrobial Advice ad hoc Expert Group (AMEG) of the European Medicines Agency (EMA), and therefore they must not be used as an empirical treatment in veterinary practice, particularly those from Category B since they are considered as critical important antimicrobials for human medicine [14]. This should also be considered for fluoroquinolones as they are also classified in this category and, in addition, very few ETEC strains were susceptible to them. In this respect, it must be mentioned that all the recommended options for NCD in the first line treatment category (Category D) such as aminopenicillins, tetracyclines and trimethoprim/sulphonamides [14] presented null or very low sensitivity rates. Taking all this into consideration, antimicrobial therapy in NCD caused by ETEC should be avoided unless there is evidence of systemic involvement. For the same reason, implementation of AST would be very useful in ETEC NCD outbreaks since it allows establishing a farm record of antimicrobial susceptibility of circulating strains, and therefore a better empirical selection of antimicrobials.

## 5. Conclusions

AMR in ETEC isolates from NCD cases in Spain is extremely high, accounting for 100% of MDR strains; these concerning results should serve as a reminder of using antibiotic treatment for NCD only in cases showing systemic involvement. Our data indicate that only a few antimicrobials (gentamicin and third- and four-generation cephalosporins) are an effective choice for the treatment of ETEC infections in diarrhoeic calves; however, the empirical use of these antibiotics should be restricted, especially third- and four-generation cephalosporins, since they are considered as critically important antimicrobials for human medicine. In addition, the observed overall lack of susceptibility of ETEC to first line antimicrobials used in veterinary medicine indicates that performing AST in each NCD outbreak is needed in order to establish an effective treatment.

## Figures and Tables

**Table 1:** Antimicrobials tested, with the minimum inhibitory concentration (MIC) breakpoints and international Standard used for each one.

Antimicrobial Family	Antimicrobial	MIC Breakpoints (mg/L)			Standard
		S	I	R	
Beta-lactam	Ampicillin	≤8		≥16	CLSI
	Amoxicillin/Clavulanic Acid	≤8		≥16	CLSI
	Cephalexin	≤4		≥8	CLSI
	Cephalothin	≤2	4	≥8	CLSI
	Ceftiofur	≤2	4	≥8	CLSI
	Cefoperazone	≤16	32	≥64	CLSI
	Cefquinome	≤2	4	≥8	CA-SFM
Aminoglycosides	Gentamicin	≤2	4	≥8	CLSI
	Neomycin	≤8	16	≥32	CA-SFM
Fluoroquinolones	Flumequine	≤8		≥16	CLSI
	Enrofloxacin	≤0.5	1	≥2	EUCAST
	Marbofloxacin	≤1	2	≥4	CLSI
Tetracyclines	Tetracycline	≤4	8	≥16	CLSI
Sulfonamides	Trimethoprim/Sulfamethoxazole	≤40		≥80	CLSI

CLSI: Clinical and Laboratory Standards Institute. CA-SFM: Comité de l’Antibiogramme de la Société Française de Microbiologie. EUCAST: European Committee on Antimicrobial Susceptibility Testing.

## Data Availability

Data are available from the corresponding author under reasonable request.
